# Co-producing a social determinants of health questionnaire for an urban population in community child health

**DOI:** 10.1136/archdischild-2020-319940

**Published:** 2021-03-03

**Authors:** Guddi Singh, Aisha Damarell

**Affiliations:** 1 Mary Sheridan Centre for Child Health, Guy's King's College and Saint Thomas' Hospitals' Medical and Dental School of King's College London, London, UK; 2 Faculty of Social Science and Public Policy, King's College London, London, UK; 3 Royal Hospital for Sick Children, Edinburgh, UK

**Keywords:** health services research, qualitative research

## Abstract

We used quality improvement (QI) and co-production methodologies to explore how child health professionals can be helped to open up conversations about poverty and other social issues in a London community child health clinic between July and October 2019.

## Abstract (precis)

In this Equipment report, Quality Improvement (QI) and co-production methodologies are used to explore how child health professionals can be helped to open up conversations about poverty in a community child health clinic. The social screening tool and resource leaflet may be adapted for other settings.

## The problem

Poverty is inextricably linked to poorer health, educational and social outcomes for children.^1^ These influences are evident in each child and family accessing community paediatric services across the country but particularly in urban areas and areas of higher deprivation, such as the Specialist Children’s and Young People Services (SCYPS) in Newham where this project was based. There are strong clinical, public health and moral grounds for paediatricians and other child health professionals identifying, preventing or mitigating the impacts of poverty and other social determinants on child health.^2^ However, questions regarding money, housing and food insecurity are not consistently or uniformly addressed in clinic, with clinicians citing awkwardness and embarrassment as common reasons for not doing so, even though parents themselves want to be asked.^3^ This is leading to missed opportunities to help families with pressing social concerns and to improve the quality of population health.

## Aim

Following on from our work looking at poverty screening tools in the acute paediatric setting,^4^ this pilot project focused on the perceived discomfort related to probing social history taking in the community. With the explicit intention of involving both service users and the wider multidisciplinary team (MDT), we aimed to co-create a refined clinical screening tool for social risk factors by October 2019. The project also aimed to explore attitudes and obstacles to discussing sensitive issues related to poverty through implementation of the screening tool in service.

## Making a case for change

In order to be able to address issues such as sensitivities around asking about social problems, and to circumvent on-the-ground logistical concerns about time and effort, this project consciously sought to involve a wide range of stakeholders from the outset. Over several weeks, we introduced a series of local child poverty teaching sessions. These were jointly conceived and delivered by a MDT composed of a paediatrician (lead author), speech and language therapist, occupational therapist and physiotherapist. Teaching sessions were voluntary but open to the entire workforce including clinical team leads, managers and patient participation representatives. These sessions were driven by and culminated in consensus that a failure to address social determinants was leading to worse outcomes for patients and they evoked a collective desire to tackle this from within the clinic. Importantly, the high levels of participation and enthusiasm arose organically and subsequent sessions were organised to garner the ideas and input of the MDT; the authors did not lead this from the top-down. These collaborative discussions resulted in a collective decision to focus on interventions or tools that could be used within the clinic to identify and address issues relating to poverty. This led to the idea to co-develop the previous work of the lead author, with a survey and resource leaflet being two of the ideas raised and discussed by the whole MDT. Thus, the case for change was ‘co-created’ by the very health professionals and patients this project aimed to help. Given this widespread support, senior clinical leads green-lighted our proposal to develop a social screening tool to help facilitate conversations about the social determinants of health with families.

## Our improvements

Screening and referral is widely invoked and well evidenced as a tool for clinicians to contribute to the mitigation of social deprivation and poverty.^5^ As many of these approaches have been developed outside the UK or for adult populations, we sought to innovate this premise to suitour service.

**Figure 1:**
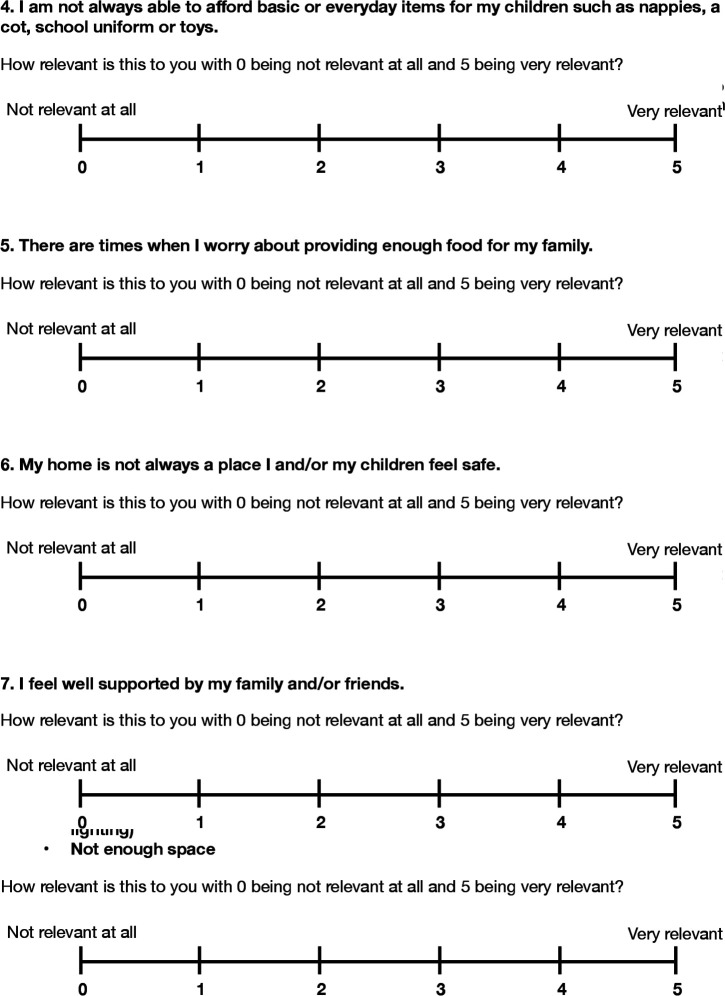
Social Determinants of Health Questionnaire (SDH-Q) for use in clinic.

**Figure 2:**
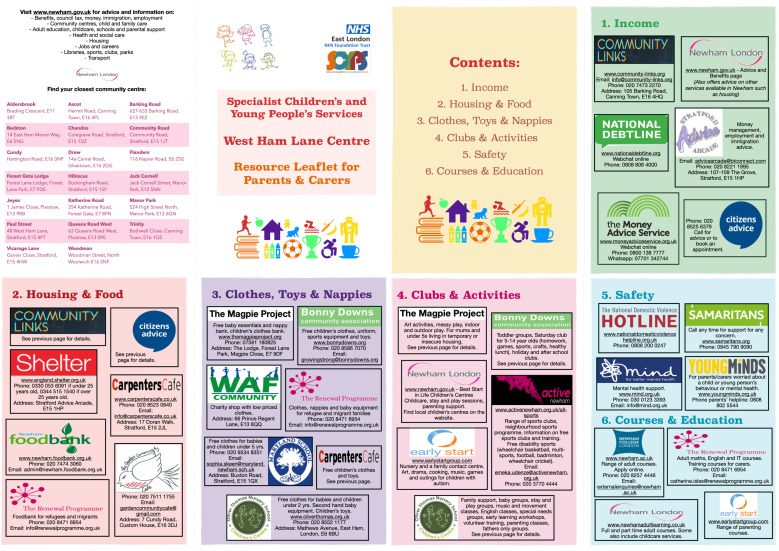
Co-produced resource pack to address social problems in Newham.

**Figure 3:**
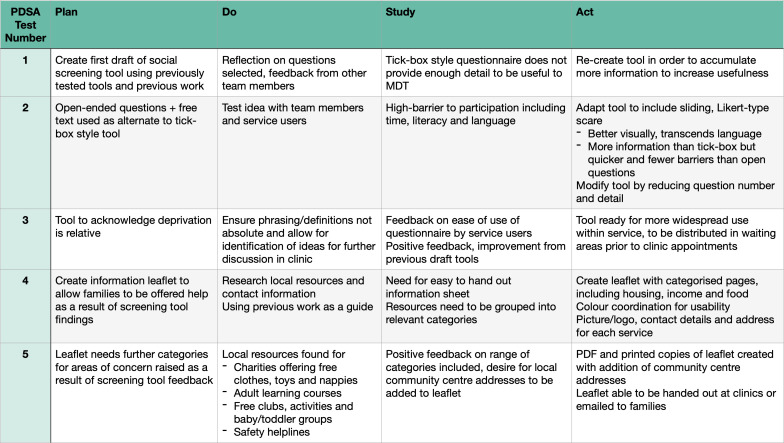
PDSA cycles demonstrating refinement of social screening tool.

Our social screening tool (figure 1) and accompanying resource leaflet (figure 2) were devised and refined via a series of Plan-Do-Study-Act (PDSA) cycles (figure 3). The leaflet was created to offer robust and tangible help to families once concerns had been elicited. Potential screening questions as well as different formats and contents for the resource leaflet were drafted, using our previous work^4^ and existing local information. Both artefacts were subsequently tested and refined to be user-friendly, non-stigmatising and relevant to both patients and clinicians.

We involved, and used qualitative feedback from, around 20 service users and 30 staff members throughout the co-production process (tables 1 and 2). Notable findings included high levels of service user eagerness to talk about their social circumstances: all service users asked were willing to complete the screening tool, and remarkably, we encountered no negative feedback. This juxtaposed with the relative lack of confidence expressed by clinicians in exploring these issues, with lack of awareness of local resources cited as a major hurdle. It was thus striking to see how effectively the introduction of a local resource pack seemed to ‘unlock’ the ability and readiness of clinicians to address social issues. Capturing this data has been crucial to learning from the pilot and planning next steps. Table 3 illustrates a case study from our project to help clinicians to see directly how this tool might play out with their patients in practice.

**Table 2:** Feedback from service users about Social Determinants of Health Questionnaire (SDH-Q)

“I know they can’t always do something but I think they should ask and know and it should be on the notes”	“These questions aren’t relevant to me at the moment but they have been in the past. It is important to be able to offer support to families in difficulty”
“Just nice to know someone cares and is thinking about these things”	“I would need help [from an interpreter] to do it”
“No point asking without offering any help”	“I’d be happy to fill it out before we came. Would be nice if the doctor could tell us places that could help”

**Table 3:** SDH-Q in action. Case study—Sam*

**The Presentation**	
Sam, aged 6 years with attention deficit hyperactivity disorder (ADHD), was referred to clinic for an autism spectrum disorder (ASD) assessment	
**Using the SDH-Q**	
Sam’s mother, Suzie, was happy to fill out the SDH-Q (see figure 1), on the basis of which her clinician was able to explore the following issues:	
**Areas of concern on SDH-Q**	**Exploration in clinic**
“Worries about not enough space at home	Suzie lives in a two-bedroom council flat with her three sons (Sam’s brothers are aged 16 months and 8 years) and her own mother. They were allocated the house when she lived with her ex-partner but the house is now too small; they do not have enough space for homework or play. Worse, Suzie struggles to find the space to help Sam with activities suggested by his therapists, and often misses appointments because she cannot afford the time or bus fare it takes to get there
“Lack of access to transport”	
“Worries about paying for housing and/or bills”	Suzie works full time in a hair salon, but her wage barely covers food and other essentials for her family. She is not always able to pay her bills, and stress about her finances prevents her from being able to fully focus on Sam’s extra needs or engage with his healthcare
“Providing enough food for the family”	
“Unable to always afford everyday items for her children”	
When asked how she felt about filling in the survey, Suzie said she felt relieved: “It’s nice that someone cares about these issues—I’ve never been asked about my living situation before! But when you think about it, it’s actually really important for my doctor to know why I struggle so much”. She felt that most advice she’d been given on managing Sam’s behaviour was simply impossible given her current situation. “I often feel like a bad mother for not being able to do what’s best for Sam”. Suzie said she would feel comfortable discussing her home situation with other healthcare professionals but that “It’s not easy to bring it up if I’m not asked about it”	
After the discussion, Suzie was given a copy of our leaflet with relevant local services highlighted. She was grateful that someone had taken the time to address her concerns and felt that the leaflet would help, as she had not heard of all of the services available	
**A few months later**	
At follow-up, Suzie told Sam’s clinician that the leaflet had prompted her to seek help. Advice from the Citizen’s Advice Bureau was already helping her to save money on housing and amenities, and visiting a clothes and toy bank meant that she could “now replace clothes that Sam is growing out of, and give him toys and books right for his age. It’s taken off a lot of pressure”. Suzie had also been receiving significant support from a local community centre, which gives access to clubs and sports for her sons. Suzie now finds she has “more time and headspace to spend with Sam” and follow healthcare professionals’ advice. Although not all of her social concerns have been relieved, Suzie was a lot more optimistic about the future, and felt more empowered to engage in Sam’s healthcare	

*‘Sam’ is a pseudonym—all names and identifying details have been changed to protect the privacy of individuals.

**Table 1:** Exemplar qualitative feedback regarding feelings about discussing social problems in clinic from both service users and clinicians

How do you feel about discussing social problems in clinic?	
(A) Service users	(B) Multidisciplinary team
“I think lots of people suffer in silence. People can’t do what the therapist suggests if they don’t have space or money for equipment. It needs to be addressed first, the stuff the therapist suggests can’t be done sometimes without the other stuff being addressed first”	“I know it is important but we are pushed for time already. Our appointments are shorter than they used to be and we need to fit the same amount into them. We just don’t have time to ask all these questions… a questionnaire would help this I suppose and then it would be easier to tell who needs help”
“It is the main issue. For us it is difficult just getting here”	“I would feel uncomfortable asking if I didn’t know how I could help them”
“It is very important for them (doctors and therapists) to know even if it is just so they can put it in their notes so people know we need extra help”	“It’s not always easy to tell who needs help. They might be dressed really well and I don’t know that they are unable to afford toys”
“We’ve been in emergency housing before and there’s no time to think about it (therapy), then we come here and they don’t know about all of that”	“I try to ask these questions in clinic anyway but having a way of identifying the most important issue for each family would make it easier”
“Doctors already struggle with having enough time”	
“It is important to ask about. They don’t affect me now but they might be in the future. I would want to know where to get help in the future”	

### Learning and next steps

Addressing the health and social burden of poverty and deprivation in health systems requires the development of novel tools and approaches. Our co-produced pilot introducing a clinical social screening tool and resource pack for use in a community child health clinic is instructive both in terms of further refinement and for adaptation for other clinical settings.

First, by allowing us to deal with both service user and provider populations’ needs simultaneously, co-production enabled us to leverage PDSA cycles to bring about the most impactful improvements to our tools. Moreover, the collaborative and egalitarian nature of using qualitative feedback to develop our tools resulted in high levels of buy-in and has also helped to distribute responsibility for sustaining the project in the longer term.

Second, the iterative and exploratory process of developing both the screening tool and the resource pack helped to bring to light, and question, assumptions about how healthcare is conventionally delivered. For instance, without careful attention, the reductive nature of healthcare questionnaires can serve to close off arenas of enquiry in the clinician–service user relationship or render responses meaningless by failing to capture what really matters to those involved. How can we guard against the mechanical or computational tendency of such tools and preserve the sanctity of the clinician–service user relationship?

Third, there were many unexpected benefits of the screening tool. For example, it became clear that parents welcomed being asked about social problems sensitively, and such enquiry was felt to occur all too rarely. One parent was moved to tears that she was asked and felt ‘cared for’ by the health system for the first time. Another parent, initially defensive and guarded in a child protection medical, opened up while using the questionnaire. He explained he did not normally feel he could admit how difficult life at home was because he was afraid his “children would be taken away”. For clinicians, the tools helped to demonstrate that their questioning was not punitive but rather humane in motivation. Moreover, clinicians felt able to ‘connect’ with their patients in ways that would not normally be possible. Finally, far from taking up extra time, the tool helped clinicians to home in on the issues most pressing for each family, thus enabling them to provide a more patient-centred or ‘bespoke’ approach to their particular problems.

**Figure 4:**
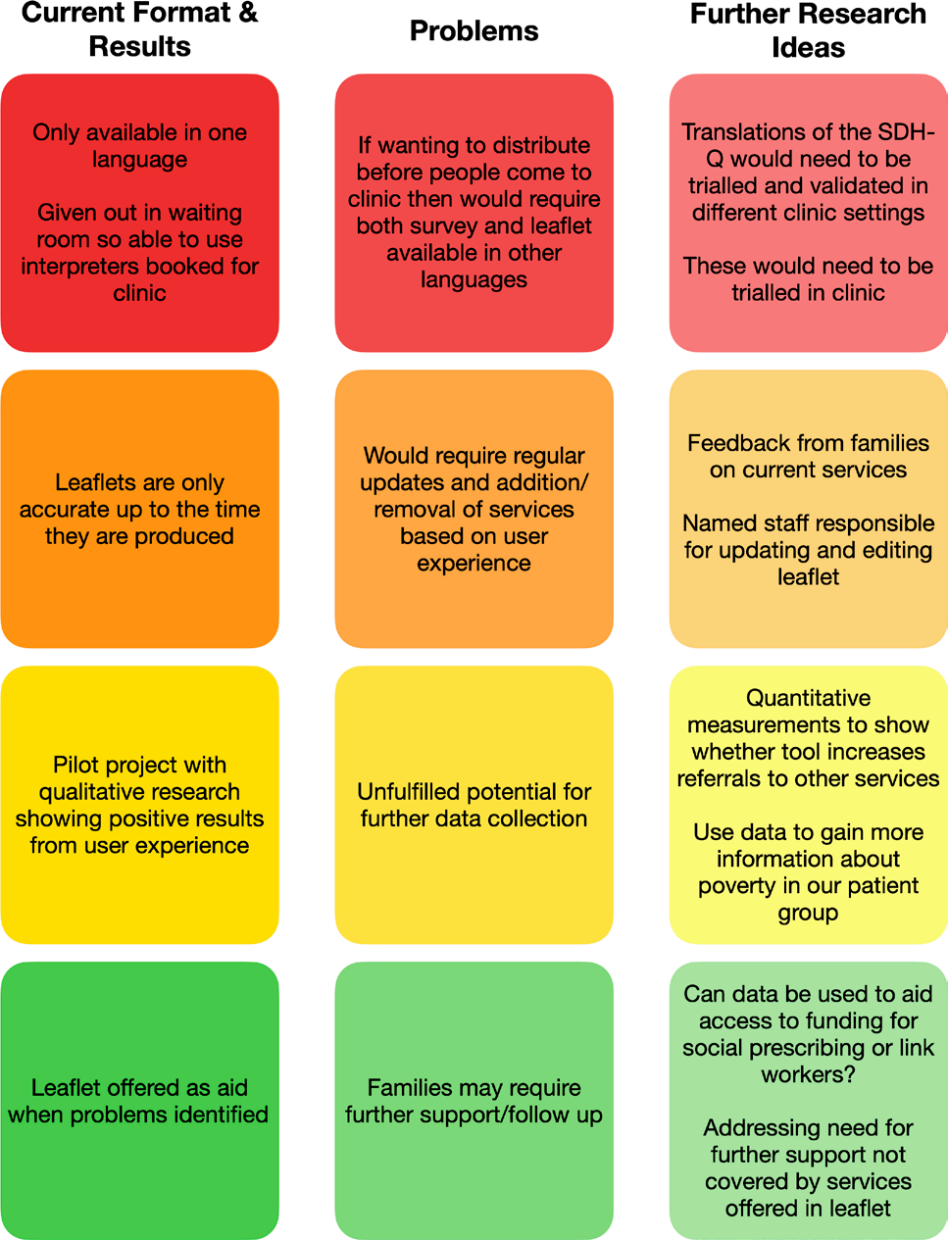
Diagram identifying areas for development.

This pilot explored if QI could be used to contend with the discomfort and resistance to asking about social problems in clinical encounters. While initial results are promising, we are aware that as a pilot study, our sample size was small; one could reasonably foresee more varied responses—including resistance or negative feedback—with larger sample sizes or in different healthcare settings. Moreover, the implications remain to be established. Could our screening tool prove helpful when used on a larger scale and more regularly? How would we meaningfully validate it? At the moment, our tool is seen as a ‘conversation opener’; however, it could conceivably be used for data collection. How can this be done without compromising trust between clinician and service user and to what use would that data be put? Can our tool work in other languages? The development of this pilot has been put on hold during the COVID-19 global pandemic, but the plan is to explore the project further with ongoing co-production locally. figure 4 lays out the potential areas for development and next steps.

The most profound and lasting lesson for the authors was the mismatch between what clinicians aspired to do for their patients and what was possible in reality. Despite deep concern about the impact of social problems on health, clinicians routinely feel hamstrung by institutional priorities and processes that pull in competing directions. This is not particular to SCYPS but rather a feature across the UK healthcare landscape.^6^ To the extent that QI can be used to help health professionals of all stripes to more closely achieve their aspirations for helping patients in challenging times, we hope our lessons prove useful to others.

## Data Availability

Data are available on reasonable request.
